# An update to the inventory of shore-fishes from the Parque Nacional Sistema Arrecifal Veracruzano, Veracruz, México

**DOI:** 10.3897/zookeys.882.38449

**Published:** 2019-10-23

**Authors:** D. Ross Robertson, Horacio Pérez-España, Omar Domínguez-Domínguez, Carlos J. Estapé, Allison Morgan Estapé

**Affiliations:** 1 Smithsonian Tropical Research Institute, Balboa, Republic of Panama Smithsonian Tropical Research Institute Balboa Panama; 2 Instituto de Ciencias Marinas y Pesquerías, Universidad Veracruzana, Hidalgo 617, Col. Río Jamapa, C.P. 94290, Boca del Río, Veracruz, México Uni­versidad Veracruzana Veracruz Mexico; 3 Laboratorio de Biología Acuática “Javier Alvarado Díaz”. Facultad de Biología, Universidad Michoacana de San Nicolás de Hidalgo. C.P. 58290. Morelia, Michoacán, México Universidad Michoacana de San Nicolás de Hidalgo Morelia Mexico; 4 150 Nautilus Drive Islamorada, Florida 33036, USA Unaffiliated Isla Morada United States of America

**Keywords:** Georeferenced aggregator records, literature review, nomenclature, observations, photography vouchers

## Abstract

Data on marine and brackish-water fishes recorded in the area of the Parque Nacional Sistema Arrecifal Veracruzano in the southwest Gulf of Mexico were extracted from online aggregators of georeferenced location records, the recent ichthyological literature reviewed, and collections and observations made to provide a more complete faunal inventory for that park. Those actions added 95 species to a comprehensive inventory published in 2013, and brought the total to 472 species, an increase of 22%. Seventy-four percent of the additions came from online aggregators of georeferenced species records, which clearly demonstrates the value of reviewing and incorporating such data into species inventories. However, different aggregators recorded different sets of species, and some of their data were linked to outdated taxonomy or included identification errors. Hence individual records from multiple aggregators need to be obtained and reviewed for such issues when using such data to compile and revise faunal inventories. Existing lists also need to be carefully reviewed to ensure that errors are not perpetuated during updates.

## Introduction

The Parque Nacional Sistema Arrecifal Veracruzano (**PNSAV**), which has an area of 522 km^2^, encompasses approximately 50 coral reefs with a combined area of 70.2 km^2^, only half of which are emergent, along a 50 km stretch of the coastline immediately adjacent to Veracruz city (~450,000 inhabitants), on the southwest coast of the Gulf of Mexico (**GoMx**). These reefs, which are situated in a shallow area of the continental shelf in which the water is < 50m deep, include some along the shoreline and others as much as 21 km offshore. This area was established as a national Marine Protected Area (**MPA**) in 1992, with modifications and additions in 2000 and 2012. As part of the management effort involved in the declaration of that MPADel Moral-Flores et al. (2013) spent five years working up a comprehensive check-list of the fish fauna of that area, was based on a review of 13 previous publications, as well as their own collections and observations. That list included 387 species of shore-fishes (marine and brackish water fishes) known from the PNSAV. There have been only two subsequent publications that provide further documentation of the PNSAV’s fish fauna, by Ayala-Rodríguez et al. (2016), and Tello-Musi et al. (2018). The present paper builds on that work by incorporating more recently available data from several sources and reviewing information in those previous publications to provide an update to that inventory.

### Materials and methods

The additions to, name changes and deletions of questionable records of some species listed from the PNSAV that are presented here are based on a review of those previous papers and incorporation of information from two additional sources: georeferenced records of species present in the PNSAV obtained from the digital databases of four major online aggregators that contain biogeographic information on fishes in the Gulf of Mexico, and our own collections and observations in the PNSAV. We reviewed and assessed the validity of the names used and questionable records of various species listed by Del Moral-Flores et al. (2013) and similarly reviewed the list of species arising from a subsequent study by Ayala-Rodríguez et al. (2016). Tello-Musi et al. (2018) provided further information on one species.

In recent years various efforts have led to large databases on the distributions of species becoming available through online museum databases, and from online aggregators that collate and distribute data from museums and a broad range of additional science sources. We took advantage of this trend by obtaining georeferenced records for species of fishes present in the area of the PNSAV from six major aggregators:

i) the Mexican National Commission for the Use and Conservation of Biodiversity (**CONABIO**: http://www.conabio.gob.mx/informacion/gis/) a national aggregator that collects data from Mexican science sources, and three aggregators that obtain data from a wider range of international sources;

ii) Integrated Digitized Biocollections (**iDigBio**: https://portal.idigbio.org/portal/search), an NSF sponsored effort run by the University of Florida that provides digital data from US collections;

iii) the Global Biodiversity Information Facility (**GBIF**: https://www.gbif.org/), which draws data from 45,000+ datasets on a broad range of organisms from a wide range sources scattered in most major areas of the globe;

iv) **Fishnet2** (http://www.fishnet2.net/), which aggregates data from ~75 museum databases in North America (mainly), Europe, Asia and Australia;

v) the Ocean Biogeographic Information System (**OBIS**: https://obis.org/), a clearing house for data that aggregates museum and local-aggregator data on various aspects of the biology of marine organisms, including their geographic distributions, is hosted in Belgium, and has 13 regional nodes scattered around the world, including the USA; and

vi) **FishBase** (http://www.fishbase.org), an international aggregator supervised by a consortium of nine non-USA international institutions that takes data on fishes in general from a broad range of sources.

These aggregators often recycle some data amongst themselves. To obtain data on occurrences of fishes from the PNSAV we searched each of those databases for georeferenced species records within a quadrat with latitudinal and longitudinal limits that closely bounded the PNSAV, with latitudes from 19.04° to 19.26°N, and longitudes from -95.75° to -96.18° W. Individual georeferenced records can be obtained from each aggregator. Since water depths within almost all of the PNSAV, particularly around the reefs, do not exceed 50m (Liaño-Carrera et al. 2019) we included in our results only those species known to occur at depths between 0–50 m in other parts of their geographic ranges. We excluded records of species of poeciliids, characids and cichlids as those are primarily or exclusively freshwater taxa. The records obtained from those aggregators were reviewed, to check for inconsistencies between putative occurrences and the known geographic ranges of species, which are not uncommon (e.g., see Robertson 2008), and to ensure included occurrences relate to updated taxonomic nomenclature, based on that in Eschmeyer’s Catalog of Fishes (Fricke et al. 2019). The PNSAV lies within the known geographic ranges of all species included in this update whose records in that MPA came from the aggregators.

Omar Domínguez-Domínguez (ODD) led a collecting expedition to the PNSAV in 2015 as part of a study of connectivity among reef fish populations throughout different reef areas in the Mexican tropical west Atlantic. That effort focused on both readily visible and small, cryptic fishes hiding in the reef matrix. For the latter the anesthetic clove oil was used to make collections (e.g., see Robertson et al. 2019). Voucher specimens of all small cryptic species collected by ODD were preserved in ethanol and have been deposited in the Colección de Peces de la Universidad Michoacana de San Nicolás de Hidalgo (curator MC Xavier Madrigal, xmguridi@yahoo.com).

Horacio Pérez-España (HP-E), based at the Universidad Veracruzana in Veracruz City, has spent decades studying reef fishes in the PNSAV. During a week in May 2019 D Ross Robertson (DRR), Carlos J Estapé (CJE), and Allison Morgan Estapé (AME) made scuba and snorkeling dives at a variety of inner and outer reefs in the northern and southern parts of the PNSAV. That activity led to the observations of species not on any previously published lists, or in online databases, and photographic records of various species.

## Results

### Changes to nomenclature used by Del Moral-Flores et al. (2013)

***Dasyatisamericana*** Hildebrand & Schroeder, 1928 and ***D.sabina*** (Lesueur, 1824) to ***Hypanusamericanus*** and ***H.sabinus***. Both species have been reassigned to the genus *Hypanus* by Last et al. (2016).

***Gymnuramicrura*** (Bloch & Schneider, 1801) to ***G.lessae***. Yokota and Carvalho 2017. Yokota and Carvalho (2017) split *G.micrura* into two species, and named the population from the Gulf of Mexico and Atlantic USA *G.lessae*, leaving *G.micrura* restricted to the coast of South America.

***Mantabirostris*** (Walbaum, 1792) to ***Mobulabirostris***. *Manta* was synonymized with *Mobula* by Last et al. (2016).

***Antennariusstriatus*** (Shaw, 1794) to ***A.scaber***. (Cuvier, 1817). *A striatus* was thought to represent a single pantropical species. However, the west Atlantic population was recently recognized as *A.scaber* (see Arnold and Pietsch 2012; Smith-Vaniz and Jelks 2014).

***Haemulonchrysargeum*** Gunther, 1859) to ***Brachygenyschrysargeum*** (Gunther, 1859). Tavera et al. (2018) reassigned this species to the newly created genus *Brachygenys*.

***Pomadasyscrocro*** (Cuvier, 1830) to ***Rhonciscuscrocro*** (Cuvier, 1830). Tavera et al. (2018) reassigned this species to the newly created genus *Rhonciscus*.

***Bairdiellaronchus*** (Cuvier, 1830) to ***Bairdiellaveraecrucis*** Jordan & Dickerson, 1908. Marceniuk et al. (2019) revised the genus and resurrected *B.veraecrucis* for the Gulf of Mexico population. *Bairdiellaronchus* is restricted to South America.

***Kyphosusincisor*** (Cuvier, 1831) to ***Kyphosusvaigiensis*** (Quoy & Gaimard, 1825). Knudsen and Clements (2013) synonymized *K.incisor* with *K.vaigiensis*.

***Stegastesvariabilis*** (Castelnau, 1855) to ***Stegastesxanthurus*** (Poey, 1860). While the name *S.variabilis* was long applied to both Brazilian and Greater Caribbean populations of what was thought to be a single species, *S.variabilis* is now considered to be a Brazilian endemic, while the Greater Caribbean population is *S.xanthurus* (Smith-Vaniz and Jelks 2014)

***Labrisomuskalisherae*** (Jordan, 1904) to ***Gobioclinuskalisherae*** (Jordan, 1904) Lin and Hastings (2013) revised the genus *Labrisomus* and split it into three, with *G.kalisherae* placed in Gobioclinus.

**Figure 1.**
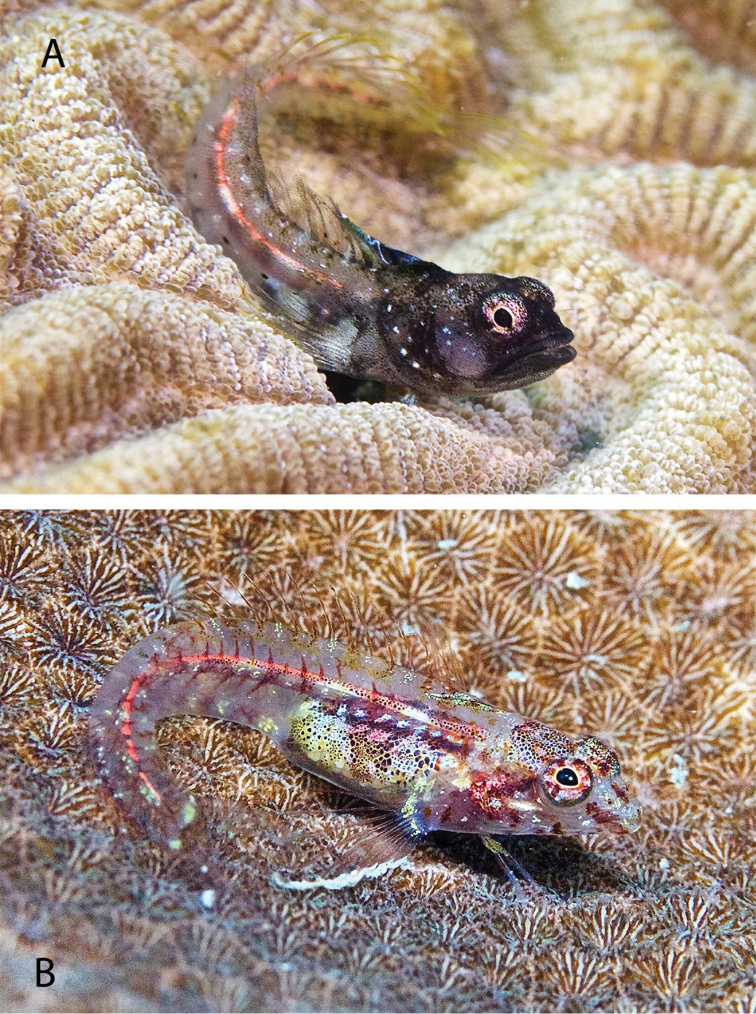
*Emblemariopsisdiaphana* at PNSAV**A** male **B** female or immature male. Photographs CJE & AME.

***Emblemariopsis* sp**. to ***Emblemariopsisdiaphana*** Longley, 1927. Photographs of this species (Figure 1) show it to be *E.diaphana*.

***Gnatholepiscauerensis*** (Bleeker, 1853) to ***Gnatholepisthompsoni*** Jordan, 1904. The species *G cauerensis* is restricted to the Indo-Pacific and St Helena in the Atlantic. *Gnatholepisthompsoni*, which is closely related to *G cauerensis*, is found on both sides of the Atlantic, including throughout the Greater Caribbean (Rocha et al. 2005; Van Tassell 2011)

### Questionable records from Del Moral-Flores et al. (2013)

Del Moral-Flores et al. (2013) listed 387 species in 206 genera and 92 families, including 21 elasmobranchs and 366 bony fishes in the PNSAV. We excluded ten species from this list that were not replaced by other names, due to likely identification errors, which would reduce the number listed by that paper to 377 species.

***Narcine* sp.** to ***Narcinebancrofti*** (Griffith & Smith, 1834). *Narcinebancrofti*, which is included in the Del Moral-Flores et al. (2013) list, is the only member of this genus currently recognized from the Gulf of Mexico and Caribbean. *Narcine* sp. may have been used due to longstanding confusion arising from misidentification of *N.bancrofti* as *N.brasiliensis* (now known to be a Brazilian endemic, see Rosa et al. 2007) or to the fact that coloration of *N.bancrofti* varies considerably. We excluded this record during the update.

***Alosasapidissima*** (Wilson, 1811) is a temperate species with a native range in eastern North America from Canada to the central east coast of Florida (Natureserve and Daniels 2019). There are only two members of the genus with established populations in the GoMx, both of which are endemic to the northern Gulf. *Alosaalabamae* Jordan & Evermann, 1896, is restricted to the northeast section of the gulf (Natureserve 2010). *Alosachrysochloris* (Rafinesque, 1820) ranges more widely, as far south as the Texas/ México border (Robertson and Caruso 2018), and is the most likely candidate for any *Alosa* found in in the southwest GoMx. Adults of *Alosa* spp. are marine, but spawn in rivers, and juveniles can be found in estuaries (Natureserve and Daniels 2019; Limburg 1996, O’Connell et al. 2004). Castro-Aguirre et al. (1999) did not record any members of this genus in estuaries on lagoons of México, which would be expected if they lived in Mexico and spawned there in rivers. The only aggregator records of any *Alosa* species in México are a few in GBIF (and Fishnet2 and FishBase) of *A.pseudoharengus* (Wilson, 1811) in Campeche, southern Veracruz state (not the PNSAV) and off the northeast tip of the Yucatan peninsula. While those Yucatan records represent misidentified *Harengulajaguana* Poey, 1865, the other records are of an *Alosa* species of uncertain identity, possibly *A.chrysochloris*, but not *A.pseudoharengus* (Hector Espinoza Pérez pers. comm. September 2019). Given that *Harengulajaguana* has been confused with *Alosa* elsewhere and is listed as present in the PNSAV by Del Moral-Flores et al. (2013), we suggest that the record of *Alosasapidissima* at the PNSAV should be viewed as *incertae sedis*. We excluded it during the update.

**Figure 2.**
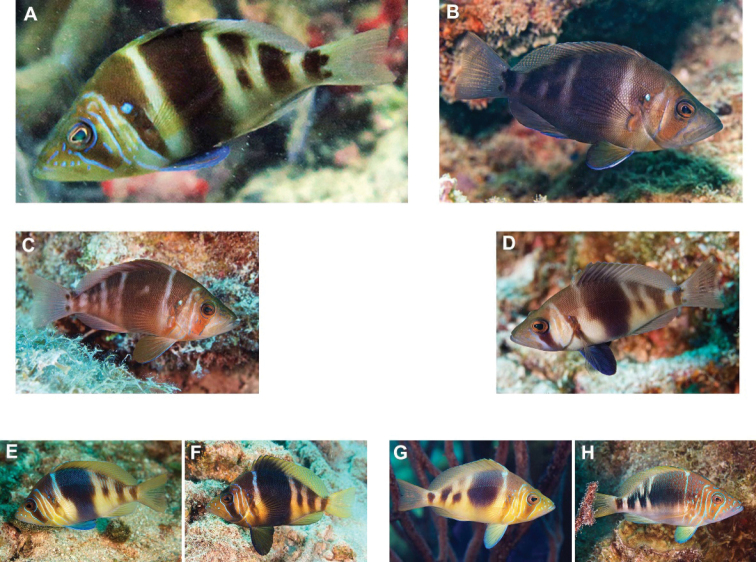
*Hypoplectrusfloridae* and *Hypoplectruspuella*. **A, B***H.floridae* from PNSAV**C, D***H.floridae* from southeast Florida **E–H***H.puella***E** Roatan **F** Bonaire **G** Bonaire **H** Southeast Florida. Photographs A HP-E, B-H CJE & AME.

***Hypoplectruspuella*** (Cuvier, 1828) to ***H.floridae*** Victor, 2012. González-Gándara et al. (2012, 2013) recorded *H.nigricans* (Poey, 1830) (a look-alike congener of the Veracruz endemic *H.atlahua* Tavera & Acero P., 2013), *H.puella* (a a look-alike congener of the species described from Florida, *H.floridae*), and *H.unicolor* (Walbaum, 1792) (a look-alike congener of the Veracruz endemic *H.castroaguirre*Del Moral-Flores et al. 2012) from reefs around Tuxpan, 250 km north of the PNSAV. Among those species Del Moral-Flores et al. (2013) listed only *H.atlahua*, *H.castroaguirre* and *H.puella* from the PNSAV. Subsequently *H.floridae* was noted from one of the Tuxpan reefs by González-Gándara (2014) and in the PNSAV by Tello-Musi et al. (2018). Given that their look-alike congeners are present in Veracruz it seems unlikely that any *H.nigricans*, *H.puella*, and *H.unicolor* also are present. Hence, just as the records of Avalos et al. (2008) of *H.nigricans* and *H.unicolor* in the PNSAV were replaced by *H.atlahua* and *H.castroaguirre*, respectively, in Del Moral-Flores et al. (2013) the *H.puella* record of Del Moral-Flores et al. (2013) from the PNSAV most likely refers to *H.floridae*. For comparison, images of *H.floridae* from the PNSAV and Florida, and of *H.puella* from the Caribbean are presented in Figure 2.

***Cynoscionjamaicensis*** (Vaillant & Bocourt, 1883). This species is largely restricted to South America and extends no further north than Honduras on the continental shoreline (Fredou and Villwock de Miranda 2015). This record most likely represents a misidentification of one of the three species of *Cynoscion* that have been found in the PNSAV, but were not included in the Del Moral-Flores et al. (2013) list (see Table 1). Castro-Aguirre et al. (1999) did not record it from México. This record was excluded during the update.

**Table 1.** Additional species of marine and brackish water fishes from the Parque Nacional Sistema Arrecifal Veracruzano not recorded by Del Moral-Flores et al. (2013). Sources: 1 CONABIO; 2 iDigBio; 3 Santander-Mosalvo et al. 2016; 4 Observations by DRR, CJE & AME; 5 Collections by ODD; 6 Ayala-Rodríguez et al. (2016); 7 GBIF; 8 Fishnet2; 9 Robertson et al. (2016a); 10 Avalos et al. (2008); 11 FishBase; 12 OBIS. Key: H = habitat; SB = soft-bottom/ estuarine, P = pelagic, R = reef, BP = benthopelagic. Distribution: WA = West Atlantic; GC = Greater Caribbean; GoMx =Gulf of Mexico; NWA = Northest Atlantic; information on global ranges and West Atlantic latitudinal ranges from https://biogeodb.stri.si.edu/caribbean/en/pages and https://www.iucnredlist.org/search

Family	Species	H	Distribution	Source
Triakidae	*Musteluscanis* (Mitchill, 1815)	SB	WA (Canada to Uruguay)	7
Potamotrygonidae	*Styracuraschmardae* (Werner, 1904)	SB	GC (GoMx to Guyana)	7
Mobulidae	*Mobulahypostoma* (Bancroft, 1831)	SB	WA (E USA to Argentina)	7
	*Mobula* spp.	P	GC (North Carolina to South Caribbean)	4
Muraenidae	*Gymnothoraxocellatus* Agassiz, 1831	SB	WA (Cuba to Brazil)	2,8
Ophichthidae	*Ahliaegmontis* (Jordan, 1884)	SB	WA (South Carolina to Brazil)	2,7,8,11,12
	*Bascanichthysbascanium* (Jordan, 1884)	SB	GC (Georgia to South Caribbean)	2,8
	*Bascanichthysscuticaris* (Goode & Bean, 1880)	SB	GC (Nth Carolina to GoMx)	7,11,12
	*Echiophisintertinctus* (Richardson, 1848)	SB	WA (North Carolina to Brazil)	7
	*Ethadophisakkistikos* McCosker & Bohlke, 1984	SB	GC (GoMx to Suriname)	2,8
	*Gordiichthysrandalli* McCosker & Böhlke, 1984	SB	GC (GoMx to South Caribbean)	2,8
	*Ophichthuscruentifer* (Goode & Bean, 1896)	SB	NWA (Maine to Suriname)	6
Congridae	*Rhynchocongerflavus* (Goode & Bean, 1896)	SB	WA (GoMx to Brazil)	2,8
	*Urocongersyringinus* Ginsburg, 1954	SB	Transatlantic (Florida to Suriname)	2,8
Engraulidae	*Anchoacubana* (Poey, 1868)	P	WA (Nth Carolina to Brazil)	2,7,8
	*Anchoalamprotaenia* Hildebrand, 1943	P	GC (GoMx to Guyana)	1,7,11,12
	*Anchoamitchilli* (Valenciennes, 1848)	P	NWA (Maine to GoMx)	1,2,6,7
	*Anchoviellaperfasciata* (Poey, 1860)	P	GC (Nth Carolina to Orinoco River)	2,7,8
	*Cetengraulisedentulus* (Cuvier, 1829)	P	WA (GoMx to Brazil)	1,2,7,8,11,12
Clupeidae	*Brevoortiagunteri* Hildebrand, 1948	P	GC (Endemic to GoMx)	2,7,8
	*Dorosomapetenense* (Günther, 1867)	P	GC (GoMx to Guatemala)	2,7
	*Opisthonemaoglinum* (Lesueur, 1818)	P	WA (Maine to Brazil)	1,6,7
Ariidae	*Cathoropsaguadulce* (Meek, 1904)	SB	GC (Endemic to GoMx)	7,8
Batrachoididae	*Opsanusbeta* (Goode & Bean, 1880)	SB	GC (E Florida to Belize)	1,2,7,11,12
Ogcocephalidae	*Dibranchusatlanticus* Peters, 1876	SB	WA (Canada to Brazil)	2,7,8
Mugilidae	*Dajausmonticola* (Bancroft, 1834)	SB	GC (North Carolina to Orinoco River)	7,8,12
	*Mugiltrichodon* Poey, 1875	SB	GC (Bermuda to South Caribbean)	2,7
Atherinopsidae	*Membrasmartinica* (Valenciennes, 1835)	P	NWA (New York to GoMx)	6
	*Menidiaberyllina* (Cope, 1867)	P	NWA (Massachusetts to GoMx)	6
Exocoetidae	*Cheilopogoncyanopterus* (Valenciennes, 1847)	P	W Atlantic & Indo-West Pacific (40°N to 40°S)	6
	*Exocoetusvolitans* Linnaeus, 1758	P	Circumtropical (35°N to 30°S)	6
	*Hirundichthysrondeletii* (Valenciennes, 1847)	P	Circumtropical (Nova Scotia to South Caribbean)	2,8,12
Hemiramphidae	*Oxyporhamphussimilis* Bruun, 1935	P	Transatlantic (40°N to 20°S)	6
Belonidae	*Strongyluramarina* (Walbaum, 1792)	P	WA (Massachusetts to Brazil)	2,6,7,8
	*Tylosurusacusacus* (Lacepéde, 1803)	P	WA (Massachusetts to Brazil)	2,7,8
Syngnathidae	*Microphislineatus* (Kaup, 1856)	SB	WA (N USA to Brazil)	7,8,11,12
	*Syngnathuslouisianae* Günther, 1870	SB	NWA (New Jersey to GoMx)	2,7,8
	*Syngnathusscovelli* (Evermann & Kendall, 1896)	SB	WA (NE Florida to Brazil)	7
Dactylopteridae	*Dactylopterusvolitans* (Linnaeus, 1758)	SB	Transatlantic (Massachusetts to Argentina)	6
Scorpaenidae	*Pteroisvolitans* (Linnaeus, 1758)	R	Indo-West Pacific; invasive	1,3,5,7
	*Scorpaenabrasiliensis* Cuvier, 1829	R	WA (Georgia to Brazil)	2,7,8
Triglidae	*Prionotusrubio* Jordan, 1886	SB	GC (North Carolina to Guyana)	2,7,8
Triglidae	*Prionotustribulus* Cuvier, 1829	SB	NWA (New York to GoMx)	1,7
Centropomidae	*Centropomusmexicanus* Bocourt, 1868	SB	WA (SE Florida to Brazil)	2,7
	*Centropomuspectinatus* Poey, 1860	SB	WA (Florida to Brazil)	2,7
	*Centropomuspoeyi* Chávez, 1961	SB	GC (SW GoMx to Belize)	2,7,8,12
Serranidae	*Hemanthiasleptus* (Ginsburg, 1952)	R	GC (North Carolina to Suriname)	1,7
	*Hypoplectrusgemma* Goode & Bean, 1882	R	GC (SE Florida to SW GoMx)	10
Apogonidae	*Apogonaurolineatus* (Mowbray, 1927)	R	GC (Georgia to South Caribbean)	6
Coryphaenidae	*Coryphaenaequiselis* Linnaeus, 1758	P	Circumtropical (Nova Scotia to Brazil)	6
Gerreidae	*Eucinostomusjonesii* (Gunther,1879)	SB	WA (Bermuda to Brazil)	2,7,8
	*Eugerresbrasilianus* (Cuvier, 1830)	SB	WA (Cuba to Brazil)	2,7,8
Haemulidae	*Haemulonboschmae* (Metzelaar, 1919)	R	GC (SW GoMx to Guyana)	4
	*Haemulonvittatum* (Poey, 1860)	R	WA (North Carolina to Brazil)	7
Sparidae	*Calamusnodosus* Randall & Caldwell, 1966	SB	GC (North Carolina to GoMx)	4
Polynemidae	*Polydactylusvirginicus* (Linnaeus, 1758)	SB	WA (North Carolina to Brazil)	2,7,8
Sciaenidae	*Cynoscionarenarius* Ginsburg, 1930	SB	GC (Endemic to GoMx)	2,7,8
	*Cynoscionnebulosus* (Cuvier, 1830)	SB	NWA (New York to GoMx)	6
	*Cynoscionnothus* (Holbrook, 1848)	SB	NWA (Chesapeake Bay to GoMx)	1,2,7,8,11,12
	*Larimusfasciatus* Holbrook,1855	SB	NWA (Massachusetts to GoMx)	2,7,8
	*Menticirrhusamericanus* (Linnaeus, 1758)	SB	WA (Massachusetts to Argentina)	1,2,7,8,11,12
	*Menticirrhuslittoralis* (Holbrook, 1847)	SB	WA (Massachusetts to Brazil)	2,7,8
	*Menticirrhussaxatilis* (Bloch & Schneider, 1801)	SB	NWA (Maine to GoMx)	2,7,8
	*Umbrinacoroides* Cuvier, 1830	SB	WA (Chesapeake Bay to Brazil)	1,2,7,8
Kyphosidae	*Kyphosuscinerascens* (Forsskal, 1775)	R	Indo-Pacific & trans-Atlantic (Bahamas to Brazil)	4
Pomacentridae	*Neopomacentruscyanomos* (Bleeker, 1856)	R	Indo-West Pacific; alien	9
Tripterygiidae	*Enneanectesboehlkei* Rosenblatt, 1960	R	GC (Florida to South Caribbean)	5
Blenniidae	*Entomacrodusnigricans* Gill, 1859	R	GC (Bermuda to South Caribbean)	4
	*Hypsoblenniushentz* (Lesueur, 1825)	R	NWA (Nova Scotia to Caribbean Mexico)	6
	*Lupinoblenniusvinctus* (Poey, 1867)	SB	GC (Cuba to South Caribbean)	12
Labrisomidae	*Gobioclinusgobio* (Valenciennes, 1836)	R	GC (Florida to South Caribbean)	2,5,7
	*Gobioclinusguppyi* (Norman, 1922)	R	GC (Florida to South Caribbean)	5
	*Paraclinusnigripinnis* (Steindachner, 1867)	R	GC (Florida to South Caribbean)	2,7,8
	*Starksiaocellata* (Steindachner, 1876)	R	GC (North Carolina to NW Caribbean)	5
Chaenopsidae	*Stathmonotushemphillii* Bean, 1885	R	GC (Bahamas to Central Caribbean)	5
Eleotridae	*Dormitatormaculatus* (Bloch, 1792)	SB	WA (North Carolina to Brazil)	7
	*Gobiomorusdormitor* Lacepede, 1800	SB	Transatlantic (Bermuda to Brazil)	7,8,11,12
Gobiidae	*Bathygobiusmystacium* Ginsburg, 1947	R	GC (Florida to South Caribbean)	2,7
	*Ctenogobiusboleosoma* (Jordan & Gilbert, 1882)	SB	WA (Chesapeake Bay to Brazil)	1,2,7,8,11,12
	*Ctenogobiusclaytonii* (Meek, 1902)	SB	GC (Endemic to GoMx)	2,7
	*Evorthoduslyricus* (Girard, 1858)	SB	WA (Chesapeake Bay to Brazil)	1,2,7,8,11,12
	*Gobioidesbroussonnetii* Lacepede, 1800	SB	WA (Georgia to Brazil)	2,7
	*Gobionellusoceanicus* (Pallas, 1770)	SB	NWA (Virginia to Suriname)	2,7
	*Neslongus* (Nichols, 1914)	R	GC (Bermuda to South Caribbean)	4
Microdesmidae	*Microdesmuscarri* Gilbert, 1966	SB	GC (GoMx to South Caribbean)	1,2,7,8,11,12
Trichiuridae	*Trichiuruslepturus* Linnaeus, 1758	BP	Transatlantic & Indo-West Pacific;	2,7,8
Xiphiidae	*Xiphiasgladius* Linnaeus, 1758	P	Circumtropical (Canada to Argentina)	11
Stromateidae	*Peprilusparu* (Linnaeus, 1758)	BP	WA (Chesapeake Bay to Argentina)	1,7
Paralichthyidae	*Citharichthysabbotti* Dawson, 1969	SB	GC (GoMx to Honduras)	1,7,11,12
	*Citharichthysmacrops* Dresel, 1885	SB	WA (Chesapeake Bay to Brazil)	1,2,7,8
	*Etropuscrossotus* Jordan & Gilbert, 1882	SB	E Pacific & W Atlantic (Virginia to Brazil)	2,7,8
Achiridae	*Achiruslineatus* (Linnaeus, 1758)	SB	WA (South Carolina to Argentina)	1,2,6,7,8,11, 12
	*Trinectesmaculatus* (Bloch & Schneider, 1801)	SB	NWA (Massachusetts to GoMx)	2,7,8
Monacanthidae	*Stephanolepissetifer* (Bennett, 1831)	R	WA (North Carolina to Brazil)	6
Tetraodontidae	*Canthigasterjamestyleri* Moura & Castro, 2002	R	GC (North Carolina to South Caribbean)	4

***Stegastesfuscus*** (Cuvier, 1830) to ***Stegastesadustus*** (Troschel, 1865). While the specific name *fuscus* was long applied to the Caribbean dusky damselfish as *Pomacentrusfuscus* under the assumption that there is a single west Atlantic species, *S.fuscus* is a Brazilian species not known to be present in the Greater Caribbean (Carter and Kaufman 2003). As *S.adustus* is in the list of Del Moral-Flores et al. (2013) we excluded this record during the update.

***Stegastespictus*** (Castelnau, 1855) to ***Stegastespartitus*** (Poey,1868). *Stegastespictus* is a Brazilian species (Carter and Kaufman 2003), juveniles of which resemble some individuals of the variably colored *S.partitus*. No other Greater Caribbean *Stegastes* species has a color pattern resembling that of *S.pictus*. The only records of *S.pictus* in the Greater Caribbean are of a few vagrants on the lesser Antilles in the southeast corner of the Caribbean, where vagrants of other species of Brazilian endemics also are known to occur. We note that *S.partitus* is in the Del Moral-Flores et al. (2013) list, and we excluded the *S.pictus* record from the update.

***Halichoerespictus*** (Poey, 1860) and ***Halichoeressocialis*** Randall & Lobel, 2003 to ***Halichoeresburekae*** Weaver & Rocha, 2007. The terminal phase male of *H.burekae* resembles that phase of both *H.pictus* and *H.socialis*, and the initial phases of *H.socialis* and *H.burekae* also are very similarly colored. Those three species form a clade within the new world *Halichoeres* species, in which *H.burekae* and *H.socialis* are sisters (Wainwright et al. 2018). *Halichoerespictus* is a conspicuous species widely distributed on reefs throughout most of the Greater Caribbean, while *H.socialis* is a Belize endemic. *Halichoeresburekae* is abundant on reefs throughout the southwest Gulf of Mexico (Aguilar-Perera and Tuz-Sulub 2009; Robertson et al. 2016a, b). The southwest gulf records of *H.pictus* and *H.socialis* predate the description date for *H.burekae* and most likely refer to that species, as there are no verified recent records of either of these two species in the southwest Gulf of Mexico since *H.burekae* was described. *Halichoeresburekae* is a common inhabitant of PNSAV reefs (our observations) that is in the Del Moral-Flores et al. (2013) list. We excluded these two records during the update.

***Ophioblenniusatlanticus*** (Valenciennes, 1836). The name *O.atlanticus* was originally applied to the populations in both the west and east Atlantic. However, the Greater Caribbean population is now recognized as *O.macclurei* (Silvester, 1915), and *O.atlanticus* refers to the east Atlantic population only (Collette et al. 2003). As the Del Moral-Flores et al. (2013) list includes *O.macclurei* as well as *O.atlanticus* the record of *O.atlanticus* was excluded during the update.

***Eleotrispisonis*** (Gmelin, 1789) to ***Eleotrisamblyopsis*** (Cope, 1871). Pezold and Cage (2002) revised the genus and found that *E.pisonis* is restricted to eastern South America. *Eleotrisamblyopsis* has been collected in the study area (see Table 1). We excluded this record when constructing the update. It should also be noted that *Eleotrisperniger* (Cope, 1871) which ranges from Veracruz south to Brazil (Pezold et al. 2015) also has aggregator records very near the PNSAV and probably occurs within it.

***Elacatinusevelynae*** (Böhlke & Robins, 1968) to ***Elacatinusprochilos*** (Böhlke & Robins, 1968), which is on the Del Moral-Flores et al. (2013) list. *Elacatinusevelynae*, which has a color pattern very similar to that of *E.prochilos*, is restricted to the Bahamas, Antilles and central Caribbean. It is not known from the northwest Caribbean. *Elacatinusprochilos* does occur along the coast of the northwest Caribbean from Honduras to northeast Yucatan and hence is the more likely of the two species to be present at Veracruz. There are no records of either species from the reefs of Campeche bank. We excluded this record from the update

**Figure 3.**
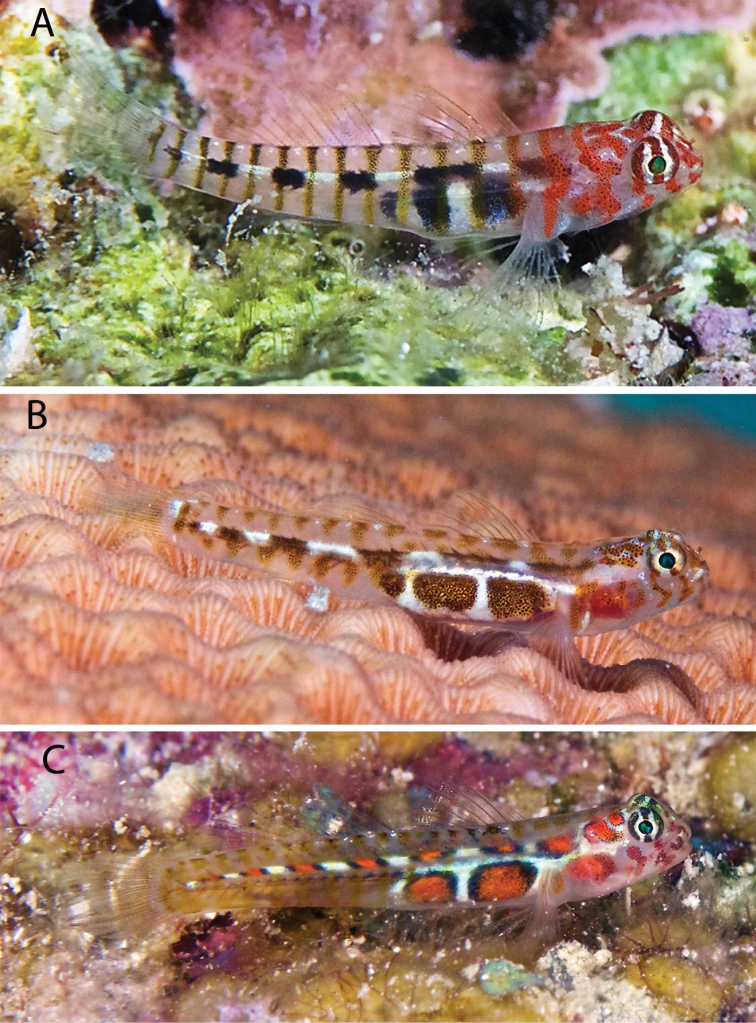
Three *Tigrigobius* species. **A***T.redimiculus* from PNSAV**B***T.saucrus* from Roatan **C***T.dilepis* from Grand Cayman. Photographs CJE & AME.

***Tigrigobiusdilepis*** (Robins & Böhlke, 1964) and ***Tigrigobiussaucrus*** (Robins, 1960) to ***Tigrigobiusredimiculus*** (Taylor & Akins, 2007). Records of *T.dilepis* and *T.saucrus* in the PNSAV precede the date of the relatively recent description of *T.redimiculus*, which was based on specimens from the PNSAV. These three species have similarly structured color patterns, with the dark marks on the head and body ranging from brown in *T.saucrus* to red in *T.dilepis* to a brown body with a red head in *T.redimiculus* (Figure 3). *Tigrigobiusredimiculus* is endemic to the southwest Gulf of Mexico, where it ranges from reefs of Veracruz state to Alacranes reef on the central Campeche Bank. The older Veracruz record is the only one for *T.dilepis* anywhere in the GoMx, while *T.saucrus* has confirmed records in the GoMx only at the Florida Keys and northern Cuba. No other species of goby in the wider Caribbean as similar to *T.redimiculus* as are *T.dilepis* or *T.saucrus* is known from the Gulf of Mexico. *Tigrigobiusredimiculus* was common on massive coral heads in very shallow water on all reefs visited, but no *T.dilepis* or *T.saucrus* (Figure 3) were observed, despite searches for them by DRR, CJE and AME in May 2019. We excluded these two records from the update.

### Questionable additional records from Ayala-Rodríguez et al. (2016)

The study of fishes in the PNSAV by Ayala-Rodriguez et al. (2016) was focused primarily on larval fishes. However, they also added 16 species, based on records of adults, that were not included by Del Moral-Flores et al. (2013), including two deep-water species (*Bregmaceroscantori* (Milliken & Houde, 1984) and *Tetragonurusatlanticus* (Lowe, 1839)) we do not include here, and three questionable records that we discuss below.

***Menidiamenidia*** (Linnaeus, 1766). The generally recognized geographic range of this species is limited to the east coast of North America, from central Florida to Newfoundland (Carpenter and Munroe 2015). This record likely relates to a congener, e.g., *M.peninsulae*, which was not recorded in the PNSAV by either Ayala-Rodríguez et al. (2016) or Del Moral-Flores et al. (2013), and the known range of which extends along the northern coast of the GoMx and south along the western coast to at least Tamiahua, 275 km from Veracruz city in the northern part of Veracruz state (Castro-Aguirre et al. 1999; Chao et al. 2015 a; Raz-Guzmán et al. 2018). The update does not include this record.

***Cynoscionregalis*** (Bloch & Schneider, 1801). The generally recognized geographic range of this species is the east coast of North America from Nova Scotia to southeast Florida, with occasional individuals on the southwest coast of Florida (Chao, 2003). *Cynoscionnebulosus* (Cuvier, 1830), which Ayala-Rodriguez et al. (2016) also recorded in the PNSAV, is a look-alike sister species that is sometimes misidentified as *C.regalis* (Chao, 2003). The known range of *C.nebulosus* extends from New York south throughout the Gulf of Mexico (except Cuba) (Chao et al. 2015b). Raz-Guzmán et al. (2018) recorded this species, but not *C.regalis*, in northern Veracruz state. Similarly, Castro-Aguirre et al. (1999) recorded *C.nebulosus* but not *C.regalis* from México. This record was not included in the update.

***Membrasvagrans*** (Goode & Bean, 1879); type locality Pensacola, Florida. Ayala-Rodriguez et al. (2016) and Raz-Guzmán et al. (2018) listed both *M.martinica* (Valenciennes, 1835) and *M.vagrans* at the PNSAV and at Tamiahua lagoon, 275 km north of the PNSAV, respectively. Castro-Aguirre et al. (1999, p. 191) treated *M.vagrans* as valid and provided a dichotomous key that separated *M vagrans* and *M.martinica* on the basis of non-overlapping numbers of anal fin rays: 14–18 for *M.vagrans* and 19–22 for *M martinica*. However, the geographic range of *M.vagrans* is overlapped completely by that of *M.martinica*, Miller (2006, p. 201) listed *M.vagrans* as a synonym of *M.martinica*, both McEachran and Fechhelm (1998, p. 886) and Robins et al. (2018, p. 185) did not include *M.vagrans* and gave anal fin ray counts for *M.martinica* of 14–21, completely overlapping the range given by Castro-Aguirre et al. (1999) for *M.vagrans*. In addition, Chernoff (1986) did not include *M.vagrans* in his revision of the Menidine silversides, and Chernoff (2003) did not include it in the FAO guide to the fishes of the northwest Atlantic. Hence it seems best at present to regard *M.vagrans* as a synonym of *M.martinica*. We did not include this record in the update.

### Additional species from the aggregators and recent literature

We found records of 95 additional species not listed by Del Moral-Flores et al. (2013) that are known to occur in depths shallower than 50 m elsewhere in their geographic ranges. Those, which include two elasmobranchs, are from 73 genera and 41 families (Table 1), with eight of those families and 42 of those genera not recorded by Del Moral-Flores et al. (2013). Seventy-one (74.7%) of the additional records came from the six aggregators. While those aggregators produced the great majority of additional records only seven species (9.9% of those in aggregator databases) were recorded in all six aggregator databases. In addition, 10 (14.1%) of those 71 species were recorded from only one aggregator, eight from GBIF and one each from FishBase and OBIS. GBIF provided the greatest number of additional aggregator records, 61 species, but missed 14.1% of species recorded by one or more of the other aggregators. CONABIO recorded 18 additional species, iDigBio 49 species, Fishnet2 42 species, FishBase 15 species, and OBIS 18 species. Given this degree of variability in numbers and identity of species recorded by different aggregators it is evident that records need to be obtained from multiple aggregators to assemble comprehensive checklists. Further, two aggregators that draw data from the same sources do not necessarily provide the same set of georeferenced records for the same species: that table shows concurrence of additional species records among those extracted from iDigBio and Fishnet2 in only 37 (69.8%) of 53 cases in which either source provided a record, with five cases of species for which records extracted directly from Fishnet2 were not present in iDigBio. In contrast, GBIF, which also receives Fishnet2 data, did record all species recorded by Fishnet2.

**Figure 4.**
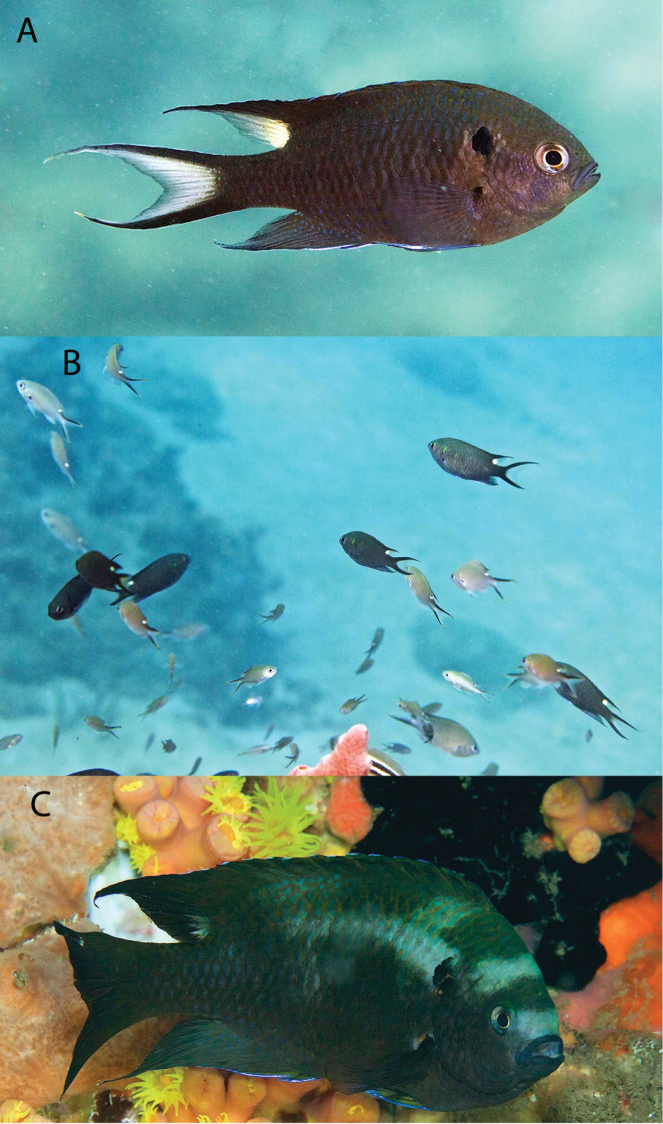
*Neopomacentruscyanomos* in the PNSAV**A** adult **B** an aggregation of large juveniles and small adults with juveniles of *Chromismultilineata***C** large male with nuptial colors. Photographs CJE & AME.

The additional species records also include 25 species not in the aggregator databases: 12 of those recorded by Ayala-Rodríguez et al. (2016), one by Avalos et al. (2008), four collected by ODD and students in 2015 (in addition to 81 species they collected that are on the Del Moral-Flores et al. (2013) list), and seven species observed (plus one previously unnamed species on the Del Moral-Flores et al. (2013) list subsequently identified), and in three cases photographed, by DRR, CJE and AME during one week of diving and snorkeling in May 2019. Additional records also include two invasive Indo-Pacific species: *Pteroisvolitans* (Linnaeus, 1758), known from the PNSAV since the beginning of 2012 (Santander-Monsalvo et al. 2012), and *Neopomacentruscyanomos* (Bleeker,1856) (see Figure 4), which was first recorded in the PNSAV by Horacio Pérez-España (HP-E) in early 2014 (see Robertson et al. 2016b). In addition, one species recorded by Tello-Musi et al. (2018) (*Hypoplectrusfloridae*) effectively replaced one of the species (*H.puella*) on Del Moral-Flores (2016) list.

The additional species added since Del Moral-Flores et al. (2013) and discussed here include species with a range of biogeographic distributions, 32 Greater Caribbean endemics (including four GoMx endemics), 13 Northwest Atlantic endemics (found in and to the north of the Greater Caribbean), 33 West Atlantic endemics found in both the Greater Caribbean and Brazil, four transatlantic species, seven circumtropical species, and two aliens from the Indo-Pacific.

### Additional species and endemics observed by the authors during May 2019

**Figure 5.**
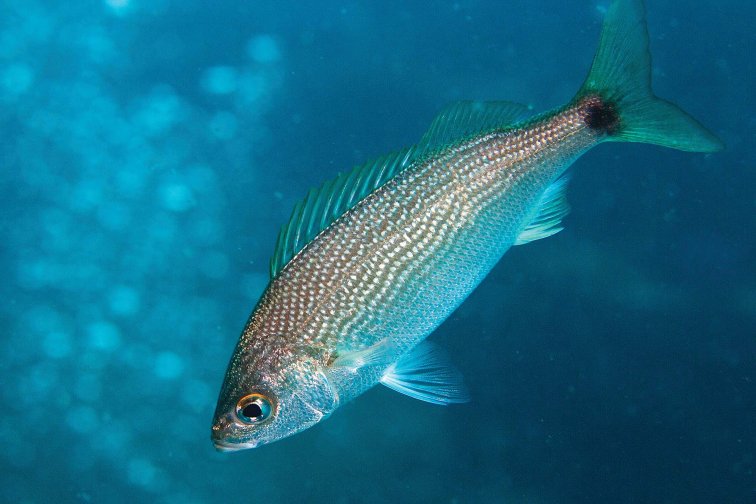
*Haemulonboschmae* in the PNSAV. Photograph CJE & AME.

**Figure 6.**
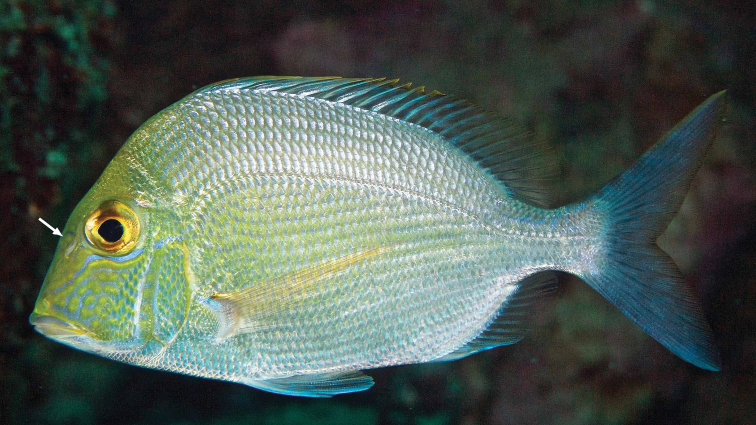
*Calamusnodosus* subadult in the PNSAV. Note the nodule (indicated by arrow) characteristic of this species on side of snout before eye. Photograph CJE & AME.

**Figure 7.**
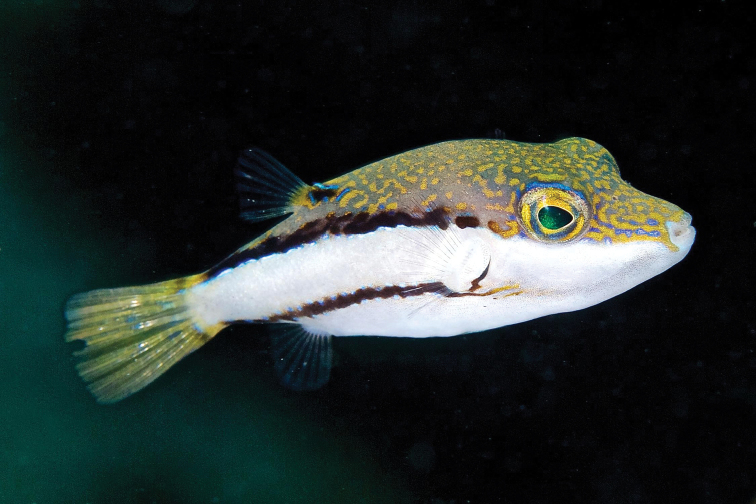
*Canthigasterjamestyleri* in the PNSAV. Photograph CJE & AME.

**Figure 8.**
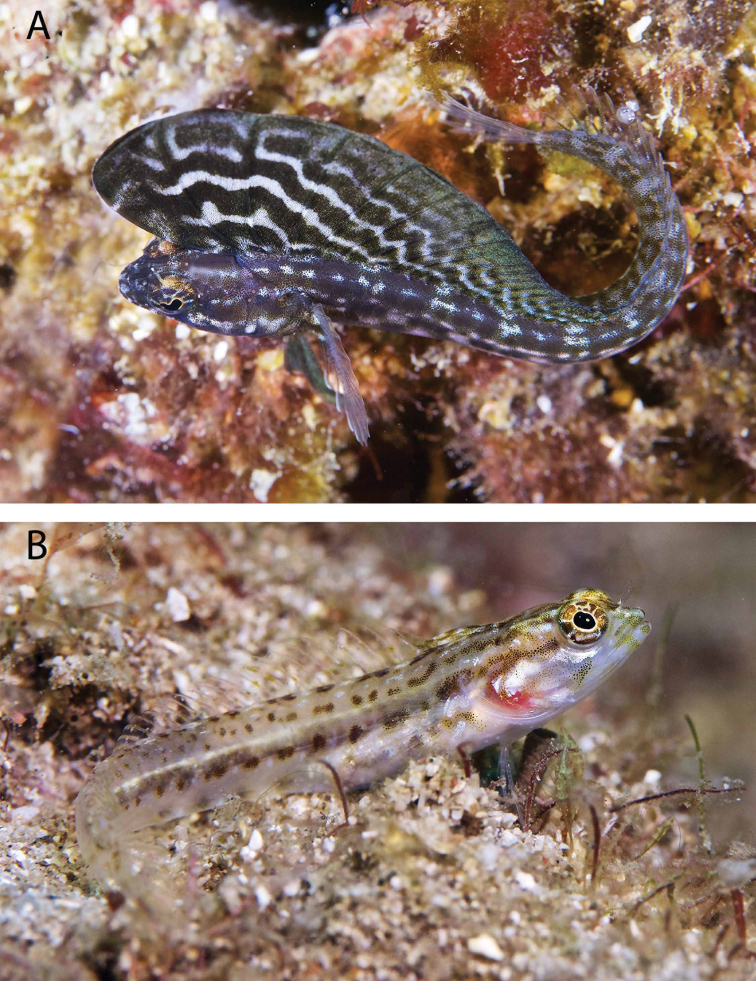
*Emblemariapandionis* in the PNSAV**A** male **B** female or uncolored male. Photographs CJE & AME.

**Figure 9.**
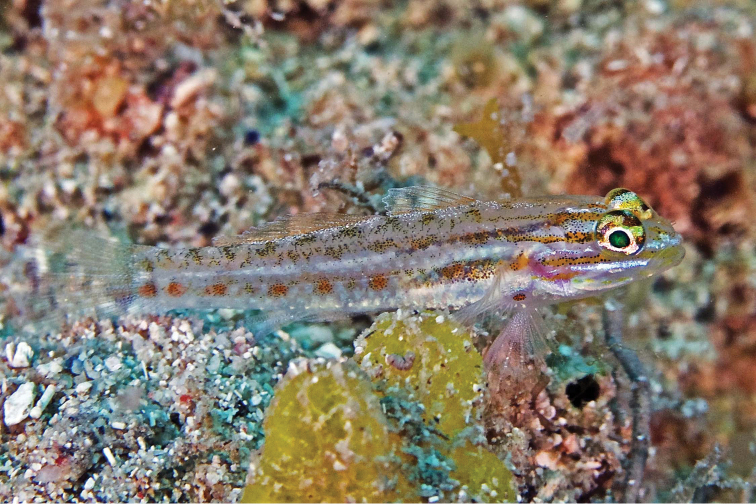
*Coryphopteruspunctipectophorus* in the PNSAV. Photograph CJE & AME.

**Figure 10.**
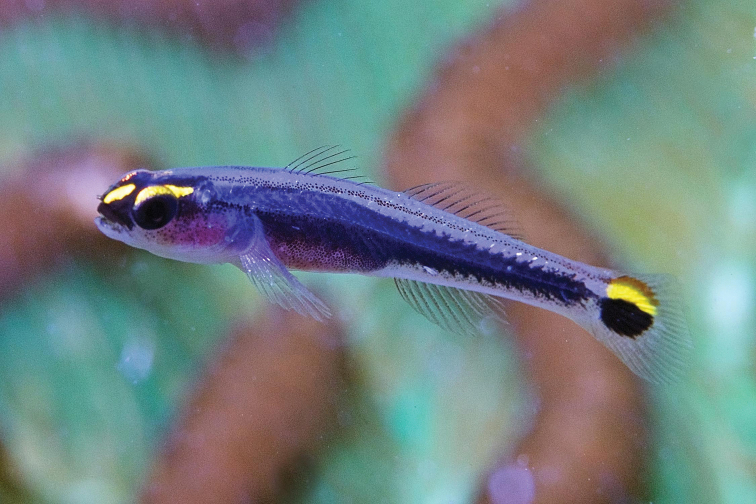
*Elacatinusjarocho* in the PNSAV. Photograph CJE & AME.

**Figure 11.**
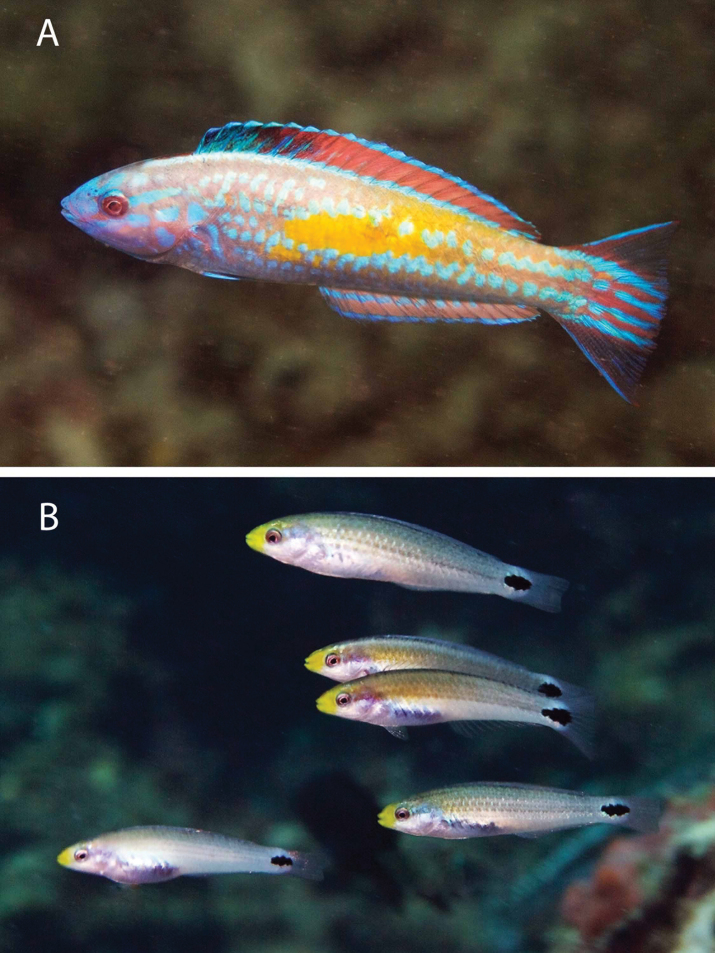
*Halichoeresburekae* in the PNSAV**A** terminal Phase male **B** initial phase individuals. Photographs CJE & AME.

*Mobulaaff.birostris* (the Caribbean manta; see Stevens et al. 2018). A large individual of this unnamed species, which has a distinctively different color pattern to that of *M.birostris* (Walbaum, 1792) (see Stevens et al. 2018), the only other morphologically similar species in the wider Caribbean, was closely observed by CJE, AME and DRR as it circled overhead during one dive; unfortunately poor visibility then did not allow for an adequate photograph. *Haemulonboschmae* (Metzelaar, 1919) was photographed by the wreck Riva Palacio (Figure 5), *Calamusnodusus* Randall & Caldwell, 1966 was photographed on De Enmedio reef (Figure 6); DRR, CJE and AME observed, and CJE photographed *Canthigasterjamestyleri* Moura & Castro, 2002 on Anegada reef (Figure 7), including one aggregation of 5 adults, in relatively shallow water for this species (14–20 m depth). H P-E had noticed this species previously on PNSAV reefs, present in some years, not in others. We repeatedly observed schools of *Kyphosus* spp. containing young adults of *Kyphosuscinerascens* (Forsskål, 1775) on several reefs which, due to its distinctly elevated dorsal and anal fins (see Knudsen and Clements 2013), is easy to distinguish from other members of the genus. DRR observed *Entomacrodusnigricans* Gill, 1859 living in barnacles in 0.5 m depth water, its typical habitat, at the base of a lighthouse on each of two emergent reefs. CJE photographed *Emblemariopsisdiaphana* Longley, 1927 (Figure 1) at Isla Verde, and Blanca reefs, *Emblemariapandionis* Evermann & Marsh, 1900 (Figure 8) on Enmedio reef, and *Coryphopteruspunctipectophorus* Springer, 1960 (Figure 9) on Anegada reef. DRR observed several pairs of *Neslongus* (Nichols, 1914), perched at the mouths of snapping-shrimp burrows in which they live, on a sand bottom with abundant live *Strombuspugilis* Linnaeus, 1758, ca. 25 m away from the base of Enmedio reef at 15 m depth. *Elacatinusjarocho* Taylor & Akins, 2007 (Figure 10), and *Halichoeresburekae* (Figure 11) both endemic to the southwest GoMx and on the Del Moral-Flores et al. (2013) list, were common and present on all reefs visited.

### Variation in coloration of two species of *Hypoplectrus* endemic to the southwest Gulf of Mexico

Two species of *Hypoplectrus* that are endemic to the southwest GoMx were recently described, both of which are present in the PNSAV. The descriptions were based on few specimens and did not adequately cover the range of variation in live coloration we have observed, and photographed, in both species at the PNSAV. As color patterns are important taxonomic aids for identifying *Hypoplectrus* species and often vary within as well as between species we present additional information on variation in both species.

**Figure 12.**
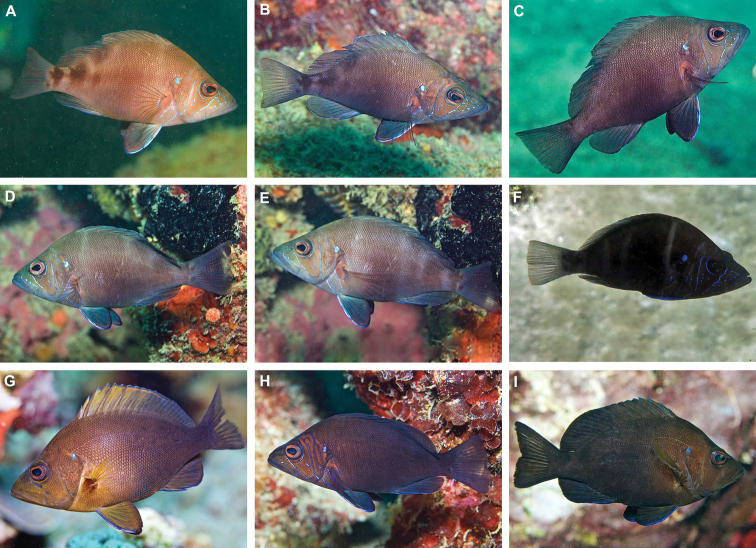
Adults of *Hypoplectrusatlahua***A–C** are of the same individual taken a few minutes apart **D, E** are of another single individual taken a few minutes apart **H** note heavy marking of blue lines on head and thin vertical blue lines on body **F** at Tuxpan, the remainder in the PNSAV. Photographs: **F** by HP-E with natural light; the remainder by CJE & AME with electronic flash.

**Figure 13.**
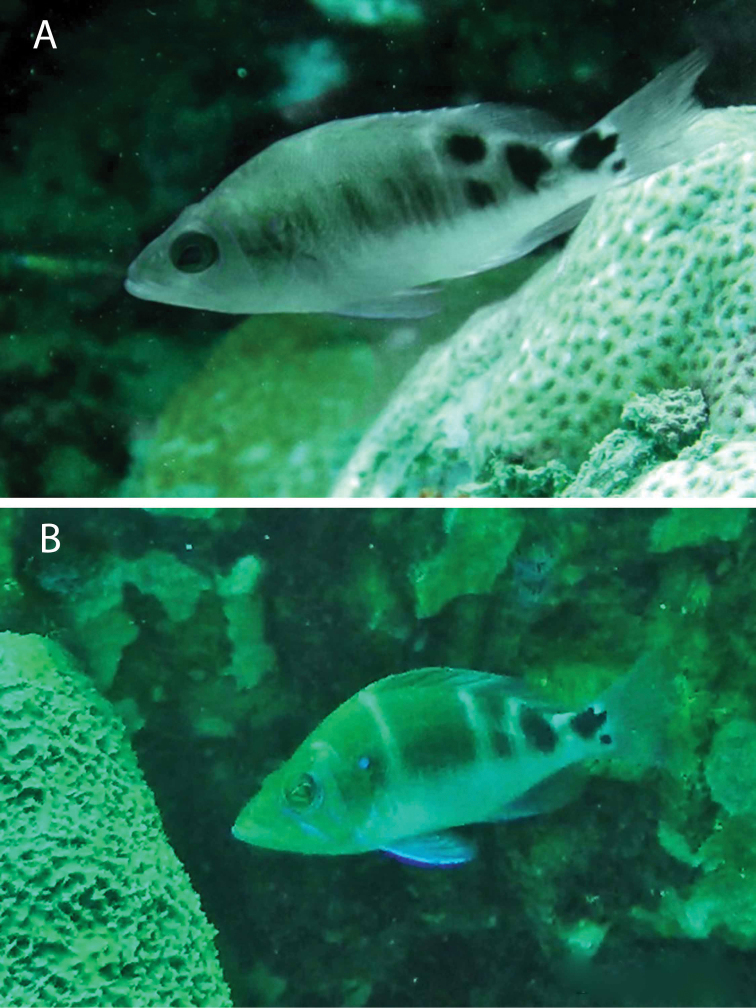
Juveniles of *Hypoplectrusatlahua***A** at PNSAV**B** at Arrecife Lobos, Tuxpan. Photographs **A** Mariana Rivera-Higueras **B** DRR. Both photographs taken with natural light.

*Hypoplectrusatlahua*. The type locality of this species is offshore from Tuxpan, 250 km north along the coast from the PNSAV. The photographs presented here represent the first published of the live coloration of this species, as the original description included only photos of freshly killed specimens. Here we present a selection to show variation in the coloration of adults and describe some of that variation. We also present images and describe the juvenile color pattern, which is quite different to that of adults. We observed a full range of color patterns from that of small juveniles grading to that of the largest adults. Large adults of *H.atlahua* have uniform dark brownish black head, body and fins, the head usually being paler than the body (Figure 12G, I). The eyes are brown, and there is a prominent blue spot at the upper corner of the operculum, varying amounts of blue lines on the face (sometimes virtually absent: Figure 13), and a prominent blue front margin to the pelvic fins (Figure 12, and see Tavera and Acero 2013). Individuals of many other species of *Hypoplectrus* often have a blue spot at the upper corner of the operculum but smaller and more weakly colored than in *H.atlahua*. There is often an indistinct darker triangular bar extending down and back from the eye to the lower rear corner of the operculum and the body can have indistinct dark bars (Figure 12F). The body sometimes has 15–20 faint vertical blue lines extending between the dorsal and ventral body profiles (Figure 12H). Adults can change color between uniform blackish brown to mid-brown with indistinct dark blotches on the rear of the body (see Figure 12A-C, all of one fish), or they may change between a dark, indistinct barred pattern and more uniform dark pattern (see Figure 12D, E, both of another single fish).

*Hypoplectrusatlahua* juveniles (Figure 13) are differently colored: juveniles sometimes have pale bodies with five dark bars on the upper body, the anterior two brown, the rear three blackish, the third bar broken into two blotches, the last bar on the end of the caudal peduncle with two black spots adhering to its rear border, each of those spots with a bright white spot above it. Alternatively they sometimes have a grey-brown body, with a darker area along the side of the head and mid-flank, and a series of black blotches at the rear of the body, a vertical pair under the anterior soft dorsal, a single blotch under the rear of the soft dorsal, a large blotch before a pair of small round spots on the end of the caudal peduncle and base of the caudal fin, with whitish areas before and behind the top of the large caudal-base blotch. The fins are translucent. As fish grow, they get a progressively darker body and fins and the rear black blotches become less distinct.

**Figure 14.**
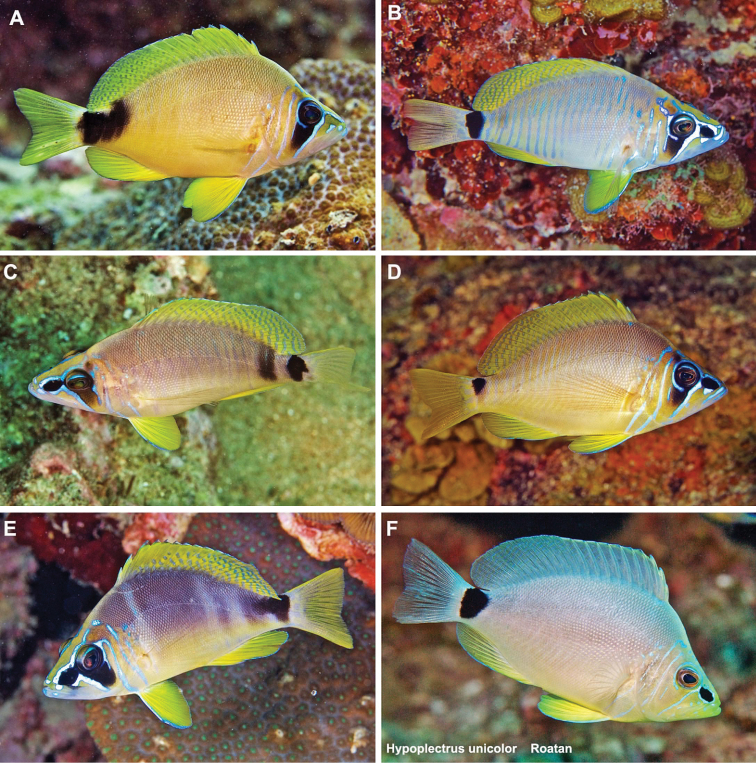
Adults of *Hypoplectruscastroaguirrei* and its Caribbean look-alike congener *H.unicolor*. **A–E***H.castroaguirrei* in the PNSAV**F***H.unicolor* at Roatan. Photographs CJE & AME.

*Hypoplectruscastroaguirrei* (Figure 14) Del Moral-Flores et al. (2011) described this species as being pale yellow, with fine blue lines on the head and chest, and blue spots on the top of the head; indistinct brown bars on the body, an oblique black bar from the top of eye down to the lower edge of the preopercle, a black blotch before the eye, both of those black marks finely edged with blue; a black blotch on the caudal peduncle; caudal, anal and pelvic fins yellow, the anal and pelvic fins with a thin blue border; the dorsal fin yellow with oblique blue lines. The type locality of this species is the PNSAV. There are very few photographs of live fish in the field available for this species (see Del Moral-Flores et al. 2011). Here we present and describe a selection taken on the reefs of the PNSAV, to provide an indication of the greater variation in this species coloration than was indicated in the original description. The ground color of the body of adults varies from pale yellowish white through mid-yellow to yellow with a brown tone over the upper body, to pale yellowish with indistinct brown bars on the upper body. The fins are yellow, and all except the caudal fin have a thin blue border. The dorsal fin, especially the soft part, is covered with many fine blue spots arranged in oblique lines, which sometimes coalesce into short, thin continuous stripes. The caudal peduncle bears a black blotch that varies considerably in size and shape, ranging from a small black blotch on the center of the upper caudal peduncle to a large, irregularly shaped blotch that covers most of the peduncle and extends forward on the rear of the body and onto the rear base of the soft dorsal fin, and sometimes is split into two separate blotches. The eye is black, surrounded by up to three black marks, including a triangular bar one angled back and down below the eye that is invariably present but varies in its length, a rounded blotch before the eye (present or absent), and a small rounded blotch above the top rear corner of the eye (present or absent). Those blotches are finely outlined with blue, there are varying amounts of blue lines on the snout, cheeks, operculum, nape, and breast, and varying arrangements of blue spots on the top of the head. The entire body of some individuals is covered with a series of ~15–20 thin vertical blue lines extending between the top and bottom profiles (Figure 14B). We have no photographs of small juveniles of this species.

## Discussion

Taking into account the reductions in the number of species recorded by Del Moral-Flores et al. (2013) and the data we present here brings the total of shore-fishes currently known in the PNSAV to 474 species, an increase of 22.5% over the total listed by Del Moral-Flores et al. (2013). These additional records also increased the number of genera of fishes in the PNSAV by 45, to 251 and the number of families by eight, to 100. Del Moral-Flores et al. (2013) used several statistical techniques to estimate the total size of that MPA fish fauna and arrived at a range of 415 to 455 species. While the highest of those estimates is close to (4.2% lower than) the adjusted currently known total number based on the data added here, the ability of experienced field observers to add seven species during one week’s snorkeling and SCUBA diving in depths of < 30 m on PNSAV reefs indicates that even 474 may represent a significant underestimate. Recently, additional shallow reefs have been discovered in and nearby to the north of PNSAV (Liaño-Carrera et al. 2019), which demonstrates the need for further studies of reefs not only of the PNSAV but elsewhere in the southwest GoMx.

Among the 95 additional species most live away from reefs, with 55.8% on and in soft bottom habitats and another 22.1% in pelagic or benthopelagic non-reef habitats. Only 22.1% of those species are demersal (or benthopelagic) forms that live on reefs and nine of those 21 species are small, cryptic fishes living within the interstices of reefs. Thus only 12 or 12.6% of the additional species represent relatively conspicuous reef fishes. Del Moral-Flores et al. (2013) efforts, in contrast were focused largely on reef fishes, mainly non-cryptic species. Populations of tropical reef-fishes and other shore-fishes do fluctuate, and rarer species may be seen at one time and not another (e.g., see comments above about *Canthigasterjamestyleri*). The update of a 50-year-old inventory of fishes on a Florida reef increased the total number of species by 21% (Starck et al. 2017), likely due to faunal changes as well as the availability of better information from sources similar to those we used here. Changes in abundances of different species likely contributed to lack of some records in the Del Moral-Flores et al. (2013) list. Furthermore, growth and increased industrial development of the city of Veracruz also may have produced changes to near-shore environments leading to changes in populations of different fish species in the PNSAV.

The Veracruz record for only seven of the additional 95 species, including four observed or collected by us, represents a significant range expansion: *Hypoplectrusgemma* Goode & Bean, 1882by 440 km (recorded on reefs of the western edge of Campeche Bank by Robertson et al. 2019); *Apogonaurolineatus* (Mowbray, 1927) by 575 km (recorded at Cayo Arenas, Campeche Bank by Robertson et al. 2019); *Kyphosuscinerascens* (Forsskål, 1775) by 440 km (recorded at Cayo Arcas on Campeche Bank by Robertson et al. 2016a); *Stathmonotushemphilii* Bean, 1885 by 440 km (recorded at Cayo Arcas by Robertson et al. 2019); and *Canthigasterjamestyleri* Moura & Castro, 2002 by 445 km (recorded at Triángulo Este reef on Campeche Bank by Robertson et al. 2019). There is little georeferenced information available on the range of the Caribbean manta, Mobulacf.birostris, with the nearest existing records to Veracruz being at the eastern tip of the Yucatan peninsula and the Flower Garden Banks, off Texas, both ~1000 km from Veracruz. Among the aggregator-additions only one record, that of *Lupinoblenniusvinctus* (Poey, 1867), represents a significant range extension, ~575 km from the west coast of the Yucatan peninsula. The fact that Veracruz is within the continental-shoreline section of the known range of all the remaining 71 additional aggregator species, almost all of which have up-to-date range maps published by https://www.iucnredlist.org, provides reason to accept those records. Judicious use of such data to update species location-lists, as we have done here, is not unusual (e.g., see Starck et al. 2017). However, while there is no reason to suspect the validity of those aggregator records we used here we cannot exclude the possibility that some are erroneous without extensive work by competent taxonomists checking specimens at a variety of museums. While such activity would be ideal it is simply not practicable in an age of shrinking resources available for basic taxonomic research at museums.

**Figure 15.**
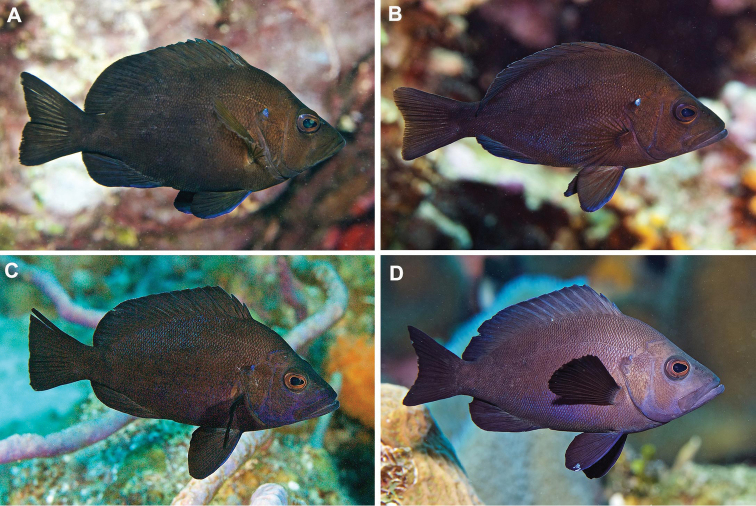
Adults of *Hypoplectrusatlahua* and its Caribbean look-alike congener *H.nigricans*. **A, B***H.atlahua* in the PNSAV**C, D***H.nigricans* at Grand Cayman and Roatan, respectively. Photographs CJE & AME.

***Hypoplectrus* species in the PNSAV**: The only confirmed all-black hamlet in Veracruz state is *H.atlahua*. *Hypoplectrusnigricans* (Poey, 1852) is the Black Hamlet from the Caribbean, Florida and Bahamas. There are minor morphometric, meristic and color differences between the two species. However, those two species belong to geographically distinct, well differentiated genetic lineages, with *H.atlahua* a member of a GoMx clade that includes *H.floridae* and *H.castroaguirre*, and *H.nigricans* (from Belize at least) belonging to a Caribbean clade (Tavera and Acero 2013). It should also be noted that *H.nigricans* from west Campeche bank reefs have a different color pattern to that of *H.atlahua* (see Robertson et al. 2016a). Adults of *H.nigricans* from the Caribbean and Florida are variable in color and some have patterns very similar to that of adult *H.atlahua*, but typically lack the strong development of fine blue lines on the head that is seen in many *H.atlahua*. What juveniles of *H.nigricans* look like from those areas is unclear. The type locality for *H.nigricans* is Havana, on the north coast of Cuba, and which clade that population belongs to (GoMx or Caribbean) and how its color relates to that of *H.atlahua* and Caribbean *H.nigricans* remains to be determined. Large adults of *H.atlahua* in some cases have coloration remarkably similarly to that of some large adults of *H.nigricans* from the Caribbean, as can be seen in Figure 15. The only difference in such cases is the larger size of the blue spot at the top corner of the operculum, and stronger blue anterior border of the pelvic fins in *H.atlahua*. Since those two allopatric, look-alike species belong to independent genetic lineages (Tavera and Acero 2013) these similarities likely are due to convergent evolution.

Tavera and Acero’s (2013) genetic analyses indicate that *H.castroaguirrei* also belongs, with *H.floridae* and *H.atlahua*, to a GoMx lineage that is well differentiated from the Caribbean lineage. As well as *H.nigricans* the Caribbean lineage includes *H.unicolor*, the name used, due to similarity in coloration, for *H.castroaguirrei* before it was recently described. Thus, as with *H.atlahua* having a color pattern that possibly evolved convergently with that of *H.nigricans*, the coloration of *H.castroaguirrei* may represent the result of independent convergent evolution by allopatric, look-alike species to a pattern that strongly resembles that of *H.unicolor*. The only consistent difference in the coloration of those two species is the presence of the strong black bar through the eye angled down towards the lower preopercle in *H.castroaguirrei* that is not seen in *H.unicolor*.

It should also be noted that Del Moral-Flores et al. (2013) listed five other species of *Hypoplectrus* as present in the PNSAV: *H.aberrans* Poey, 1868, *H.chlorurus* (Cuvier, 1828), *H.gumigutta* (Poey, 1851), *H.guttavarius* (Poey, 1852), and *H.indigo* (Poey, 1851). DRR, CJE and AME did not observe any of these in May 2019 and we are not aware of any photographs of them from PNSAV that could be reviewed. Many species in this genus exhibit individual variation in coloration (see images in Robertson and Van Tassell 2015). The color patterns of some individuals of *H.aberrans*, *H.gumigutta* and *H.guttavarius*, all of which do or can have large areas of yellow on the body, resemble the coloration of some individuals of *H.castroaguirre*, which, as can be seen in Figure 14, varies in color. Similarly, the coloration of *H.aberrans* resembles that of a *H.atlahua* with a pale tail, and the coloration of *H.indigo* resembles that of *H.floridae* with the addition of heavy blue overtones. Revision of images of live individuals of those five species taken in the PNSAV would be useful for clarifying exactly how many species of this genus actually occur in the PNSAV.

## Conclusions

Comprehensive inventories of local to regional fish faunas require not only literature reviews augmented by field observations and collections by inventory authors, but also careful and comprehensive review of information available in the databases of online aggregators. Those aggregators draw data from a variety of sources and provide information from museums that catalog specimens obtained since the beginning of research on fishes. Much of the aggregator material only became available recently and the amount of legacy information the aggregators provide continues to increase. Review of such material, and our own observations and collections, increased by 22% the known fish fauna of a large MPA next to a city with a substantial population and a university that has sponsored research on those fishes over the past several decades. This demonstrates the value of such aggregator material. However, different aggregators provide different information and multiple aggregators need to be consulted to obtain the fullest picture of their information. Aggregators do not themselves correct errors in material emanating from the primary sources of their information, which invariably contain uncorrected errors. Limitations in the quality of aggregator information due to misidentifications, outdated taxonomy and nomenclature, and errors in georeferencing of species records must be taken into consideration when using such data. In addition, the content of older lists needs to be carefully reviewed when updating faunal lists, to help ensure that old errors do not continue to be perpetuated, and that updates do not consist solely of additions to faunas.

## References

[B1] Aguilar-Perera A Tuz-Sulub A (2009) Occurrence of the Mardi Gras wrasse, *Halichoeresburekae* (Teleostei: Labridae) in the Alacranes Reef, off northern Yucatán Peninsula. Zootaxa 2298: 64–68. 10.11646/zootaxa.2298.1.5

[B2] Arnold RJ Pietsch TW (2012) Evolutionary history of frogfishes (Teleostei: Lophiiformes: Antennariidae): a molecular approach. Molecular Phylogenetics and Evolution 62: 117–129. 10.1016/j.ympev.2011.09.012 21985964

[B3] Avalos MAR Jordan LKB Walker BK Gilliam DS Hinojosa EC Speiler RE (2008) Fish and Coral Reef Communities of the Parque Nacional Sistema Arrecifal Veracruzano (Veracruz Coral Reef System National Park) Veracruz, México: Preliminary Results. Proceedings of the 60^th^ Gulf and Caribbean Fisheries Institute November 5–9, 2007 Punta Cana, 427–435.

[B4] Ayala-Rodríguez GA Ordóñez-López U Meiners C Marín-Hernández M (2016) Listado taxonómico, aspectos ecológicos y biogeográficos de las larvas de peces del Sistema Arrecifal Veracruzano, Suroeste del Golfo de México (junio 2011-junio 2013). Revista de Biología Marina y Oceanografía 51: 255–264. 10.4067/S0718-19572016000200004

[B5] Carpenter KE Munroe T (2015) *Menidiamenidia*. The IUCN Red List of Threatened Species 2015: e.T16441575A16510092. 10.2305/IUCN.UK.2015-4.RLTS.T16441575A16510092.en

[B6] Carter JA Kaufman L (2003) Pomacentridae. In: Carpenter KE (Ed.) The living marine resources of the Western Central Atlantic. Volume 3: Bony fishes part 2 (Opistognathidae to Molidae). FAO species identification guide for fishery purposes and American Society of Ichthyologist and Herpetologists Special Publication No. 5. FAO, Rome.3: 1694–1700.

[B7] Castro-Aguirre JL Espinosa-Pérez H Schmitter-Soto JJ (1999) Ictiofauna estuarino-lagunar y vicaria de México. Noriega-Limusa, IPN, Mexico City, 711 pp.

[B8] Chao L Vega-Cendejas M Jelks H Tolan J Espinosa-Pérez H (2015a) *Menidiapeninsulae*. The IUCN Red List of Threatened Species 2015: e.T155207A70180472.

[B9] Chao L Espinosa-Pérez H Barbieri L (2015b) *Cynoscionnebulosus*. The IUCN Red List of Threatened Species 2015: e.T193266A49237289. 10.2305/IUCN.UK.2015-2.RLTS.T193266A49237289.en

[B10] Chao L (2003) Sciaenidae. In: Carpenter KE (Ed.) The living marine resources of the Western Central Atlantic. Volume 3: Bony fishes part 2 (Opistognathidae to Molidae). FAO species identification guide for fishery purposes and American Society of Ichthyologist and Herpetologists Special Publication No. 5. FAO, Rome.3: 1583–1653.

[B11] Chernoff B (1986) Phylogenetic relationships and reclassification of Menidiinae silverside fishes with emphasis on the tribe Membradini. Proceedings of the Academy of Natural Sciences of Philadelphia, 138: 189–249.

[B12] Chernoff B (2003) Atherinidae and Atherinopsidae. In: Carpenter KE (Ed.) The living marine resources of the Western Central Atlantic. Volume 3: Bony fishes part 2 (Opistognathidae to Molidae). FAO species identification guide for fishery purposes and American Society of Ichthyologist and Herpetologists Special Publication No. 5. FAO, Rome.2: 1086–1103.

[B13] Collette BB Williams JT Thacker CE Smith MJ (2003) Shore fishes of Navassa Island, West Indies: a case study on the need for rotenone sampling in reef fish biodiversity studies. Aqua, Journal of Ichthyology and Aquatic Biology 6: 89–131.

[B14] Del Moral-Flores LF Tello-Musi JL Martínez-Pérez JA (2012) Descripción de una nueva especie del género *Hypoplectrus* (Actinopterigy (sic)): Serranidae del Sistema Arrecifal Veracruzano, suroeste de Golfo de México. Revista de Zoología, Universidad Nacional Autónoma de México 22: 1–10.

[B15] Del Moral-Flores L Tello-Musi J Reyes-Bonilla H Pérez-España H Martínez-Pérez J Horta-Puga G Velazco-Mendoza L Álvarez del Castillo-Cárdenas P (2013) Lista sistemática y afinidades zoogeográficas de la ictiofauna del Sistema Arrecifal Veracruzano, México. Revista Mexicana de Biodiversidad 84: 825–846.

[B16] Fredou FL Villwock de Miranda L (2015) *Cynoscionjamaicensis*. The IUCN Red List of Threatened Species 2015: e.T47147457A49237421. 10.2305/IUCN.UK.2015-2.RLTS.T47147457A49237421.en

[B17] Fricke R Eschmeyer WN Van der Laan R (Eds) (2019) Eschmeyer’s Catalog Of Fishes: Genera, Species, References. http://researcharchive.calacademy.org/research/ichthyology/catalog/fishcatmain.asp [Accessed June 2019]

[B18] González-Gándara C (2014) Peces de Arrecife Blake, Veracruz, México: Inventario, distribución y afinidades zoogeográficas. Ecosistemas y Recursos Agropecuarios 2: 87–97.

[B19] González-Gándara C de la Cruz FV Salas Pérez Domínguez Barradas C (2012) Lista de los peces de Tuxpan, Veracruz, México. Revista Científica UDO Agrícola 12: 675–689.

[B20] González-Gándara C Lozano Vilano M de L de la Cruz FV Domínguez Barradas C (2013) Peces del sistema arrecifal Lobos-Tuxpan, Veracruz, México. Universidad y Ciencia 28: 191–208.

[B21] Knudsen SW Clements KD (2013) Revision of the family Kyphosidae (Teleostei: Perciformes). Zootaxa 3751: 1–101. 10.11646/zootaxa.3751.1.1 29097648

[B22] Last PR Naylor GJP Manjaji-Matsumoto BM (2016) A revised classification of the family Dasyatidae (Chondrichthyes: Myliobatiformes) based on new morphological and molecular insights. Zootaxa 4139: 345–368. 10.11646/zootaxa.4139.3.2 27470808

[B23] Liaño-Carrera F Camarena-Luhrs T Gómez-Barrero A Martos-Fernández FJ Ramírez-Macías JI Salas-Monreal D (2019) New coral reef structures in a tropical coral reef system. Latin American Journal of Aquatic Research 47: 270–281. 10.3856/vol47-issue2-fulltext-7

[B24] Limburg KF (1996) Modelling the ecological constraints on growth and movement of juvenile American Shad (*Alossasapidissima*) in the Hudson River estuary. Estuaries 19: 794–813. 10.2307/1352298

[B25] Lin H-C Hastings PA (2013) Phylogeny and biogeography of a shallow water fish clade (Teleostei: Blenniiformes). BMC Evolutionary Biology 13: 1–18. 10.1186/1471-2148-13-210 24067147 PMC3849733

[B26] Marceniuk AP Molina EG Caires RA Rotundo MM Wosiacki WB Oliveira C (2019) Revision of *Bairdiella* (Sciaenidae: Perciformes) from the western South Atlantic, with insights into its diversity and biogeography. Neotropical Ichthyology 17: e180024: 1–18. 10.1590/1982-0224-20180024

[B27] McEachran JD Fechhelm JD (1998) Fishes of the Gulf of Mexico. Volume 1: Myxiniformes to Gasterosteiformes. University of Texas Press, Austin, 1112 pp.

[B28] Miller RR (2006) Freshwater Fishes of México. University of Chicago Press, Chicago, 490 pp.

[B29] NatureServe (G. Hammerson) (2010) . *Alosaalabamae*. The IUCN Red List of Threatened Species 2010: e.T908A13094078. 10.2305/IUCN.UK.2010-3.RLTS.T908A13094078.en

[B30] NatureServe Daniels A (2019) *Alosasapidissima*. The IUCN Red List of Threatened Species 2019: e.T191206A82664336. 10.2305/IUCN.UK.2019-2.RLTS.T191206A82664336.en

[B31] O’Connell M Cashner R Schieble C (2004) Fish assemblage stability over fifty years in the Lake Pontchartrain estuary; comparisons among habitats using Canonical Correspondence Analysis. Estuaries 27: 807–817. 10.1007/BF02912042

[B32] Pezold FL Cage B (2002) A review of the spinycheek sleepers, genus *Eleotris* (Teleostei: Eleotridae), of the Western Hemisphere, with comparisons to the west African species. Tulane Studies in Zoology and Botany 31: 19–63.

[B33] Pezold F van Tassell J Aiken KA Tornabene L Bouchereau J-L (2015) *Eleotrisperniger*. The IUCN Red List of Threatened Species 2015: e.T185990A1799642. 10.2305/IUCN.UK.2015-2.RLTS.T185990A1799642.en

[B34] Randall JE Lobel PS (2003) *Halichoeressocialis*: a new labrid fish from Belize. Copeia 2003: 124–130. 10.1643/0045-8511(2003)003[0124:HSANLF]2.0.CO;2

[B35] Raz-Guzmán A Huidobro L Padilla V (2018) An updated checklist and characterization of the ichthyofauna (Elasmobranchii and Actinopterygii) of the laguna de Tamianhua, Veracruz, México. Acta Ichthyologica et Piscatoria 48: 341–362. 10.3750/AIEP/02451

[B36] Robertson DR (2008) Global biogeographic databases on marine fishes: caveat emptor. Diversity and Distributions. 14: 891–892. 10.1111/j.1472-4642.2008.00519.x

[B37] Robertson DR Caruso J (2018) *Alosachrysochloris* (errata version, 2019). The IUCN Red List of Threatened Species 2018: e.T196673A143863055. 10.2305/IUCN.UK.2018-2.RLTS.T196673A143863055.en

[B38] Robertson DR Pérez-España H Nuñez Lara E Puc Itza F Simoes N (2016a) The fishes of Cayo Arcas (Campeche Bank, Gulf of Mexico): an updated checklist. ZooKeys 640: 139–155. 10.3897/zookeys.640.10862 PMC5240370 28138290

[B39] Robertson DR Simoes N Gutiérrez Rodríguez C Piñeros VJ Pérez-España H (2016b) An Indo-Pacific damselfish widely established in the southwest Gulf of Mexico: prospects for a wider, adverse invasion. Journal of the Ocean Science Foundation 19: 1–17. 10.5281/zenodo.44898

[B40] Robertson DR Domíinguez-Domínguez O Lopez Arollo YM Moreno Mendoza R Simoes N (2019) Reef-associated fishes from the offshore reefs of western Campeche Bank, Mexico, with a discussion of mangroves and seagrass beds as nursery habitats. ZooKeys 843: 71–115. 10.3897/zookeys.843.33873 31139001 PMC6522471

[B41] Robertson DR Van Tassell J (2015) Shorefishes of the Greater Caribbean: online information system. Version 1.0 Smithsonian Tropical Research Institute, Balboa, Panamá. https://biogeodb.stri.si.edu/caribbean/en/pages

[B42] Robins RH Page LM Williams JD Randall ZS Sheehy GE (2018) Fishes in the Fresh Waters of Florida: an Identification Guide and Atlas. University of Florida Press, Gainesville, 468 pp.

[B43] Rocha LA Robertson DR Rocha CR van Tassell JL Craig MT Bowen BW (2005) Recent invasion of the tropical Atlantic by an Indo-Pacific coral reef fish. Molecular Ecology 14: 3921–3928. 10.1111/j.1365-294X.2005.02698.x 16262848

[B44] Rosa RS Furtado M (2007) *Narcinebrasiliensis*. The IUCN Red List of Threatened Species 2007: e.T63157A12602819. 10.2305/IUCN.UK.2007.RLTS.T63157A12602819.en

[B45] Santander-Monsalvo J López-Huerta I Aguilar-Perera A Tuz-Sulub A (2012) First record of the red lionfish (*Pteroisvolitans* [Linnaeus, 1758]) off the coast of Veracruz, México. BioInvasions Records 1: 121–124. 10.3391/bir.2012.1.2.07

[B46] Smith-Vaniz W Jelks HL (2014) Marine and inland fishes of St. Croix, U. S. Virgin Islands: an annotated checklist. Zootaxa 3803: 1–120. 10.11646/zootaxa.3803.1.1 24871150

[B47] Starck WA Estapé CJ Morgan Estapé A (2017) The fishes of Alligator Reef and environs in the Florida Keys: a half-century update. Journal of the Ocean Science Foundation 7: 74–117. 10.5281/zenodo.851651

[B48] Stevens GFD Dando M Notabartolo di Sciara G (2018) Guide to Manta and Devil Rays of the World. Princeton University Press, Princeton, 144 pp.

[B49] Tavera J Acero P A (2013) Description of a new species of Hypoplectrus (Perciformes: Serranidae) from the southern Gulf of Mexico. Aqua 19: 1–21.

[B50] Tavera J Acero PA Wainwright PC (2018) Multilocus phylogeny, divergence times, and a major role for the benthic-to-pelagic axis in the diversification of grunts (Haemulidae). Molecular Phylogenetics & Evolution 121: 212–223. 10.1016/j.ympev.2017.12.032 29307507

[B51] Tello-Musi JL Chávez-Arteaga M Cruz-López FdeJ Martínez-Pérez JA (2018) Adenda a la lista sistemática y afinidades zoogeográficas de la ictiofauna del Sistema Arrecifal Veracruzano, México. Revista de Zoológia 29: 81–83.

[B52] Van Tassell JL (2011) Gobiiformes of the Americas. In: Patzner RA Van Tassell JL Kovacic M Kapoor GG (Eds) The Biology of Gobies.CRC Press, Boca Raton, 139–176. 10.1201/b11397-14

[B53] Wainwright PP Santini F Bellwood D Robertson DR Rocha L Alfaro M (2018) Phylogenetics and geography of speciation in new world Halichoeres wrasses. Molecular Phylogenetics and Evolution 121: 35–45. 10.1016/j.ympev.2017.12.028 29289544

